# Achilles tendon allograft for an irreparable massive rotator cuff tear with bony deficiency of the greater tuberosity

**DOI:** 10.1007/s00167-016-3989-1

**Published:** 2016-01-25

**Authors:** Alexandre Lädermann, Patrick J. Denard, Sophie Abrassart, Adrien J.-P. Schwitzguébel

**Affiliations:** 1 Division of Orthopaedics and Trauma Surgery, La Tour Hospital, Rue J.-D. Maillard 3, 1217 Meyrin, Switzerland; 2 Faculty of Medicine, University of Geneva, Rue Michel-Servet 1, 1211 Geneva, Switzerland; 3 Division of Orthopaedics and Trauma Surgery, Department of Surgery, Geneva University Hospitals, Rue Gabrielle-Perret-Gentil 4, 1211 Geneva, Switzerland; 4 Southern Oregon Orthopedics, Medford, OR USA; 5 Department of Orthopaedics and Rehabilitation, Oregon Health and Science University, Portland, OR USA

**Keywords:** Massive rotator cuff, Bone and tendon insufficiency, Achilles allograft, Treatment, Outcome, Function

## Abstract

Management of combined bony and tendinous deficiency of the posterosuperior rotator cuff represents a challenge in young patients. In this case report, a 44-year-old woman that presented an osteonecrosis of the greater tuberosity had a pseudoparalytic shoulder. She beneficiated from a fresh-frozen Achilles tendon allograft with calcaneal bone, which was used to reconstruct the rotator cuff and the concomitant bony defect. At 12-month follow-up, the patient was pain free and had complete range of motion, normal strength, a SANE score of 95 and radiographically the allograft was healed. An Achilles tendon allograft may therefore be a viable surgical option to reconstruct a combine posterosuperior rotator cuff tear and greater tuberosity bone defect.

*Level of evidence* IV.

## Introduction

Nonunion or malunion of the greater tuberosity following proximal humerus fracture can result in substantial functional disability. In old patients, this combined bony and rotator cuff deficiency can be easily managed with reverse shoulder arthroplasty. However, it is not a satisfactory option in young patients due to the long-term complication and survival rate of reverse shoulder arthroplasty [2, 6, 11]. Bone and tendon allografts are an option in the setting of combined bony and tendon deficiency and have been described the elbow and knee for example [5]. The present case report describes an original technique of rotator cuff and greater tuberosity reconstruction with Achilles tendon allograft which could represent an alternative to arthroplasty in a young patient with greater tuberosity and rotator cuff deficiency. To the best of our knowledge, this is the first time that this procedure has been proposed for this problem. The particular interests of Achilles tendon and calcaneus allograft are the robustness of the tendinous component and the malleability of the bony component.

## Case report

**Fig. 1:**
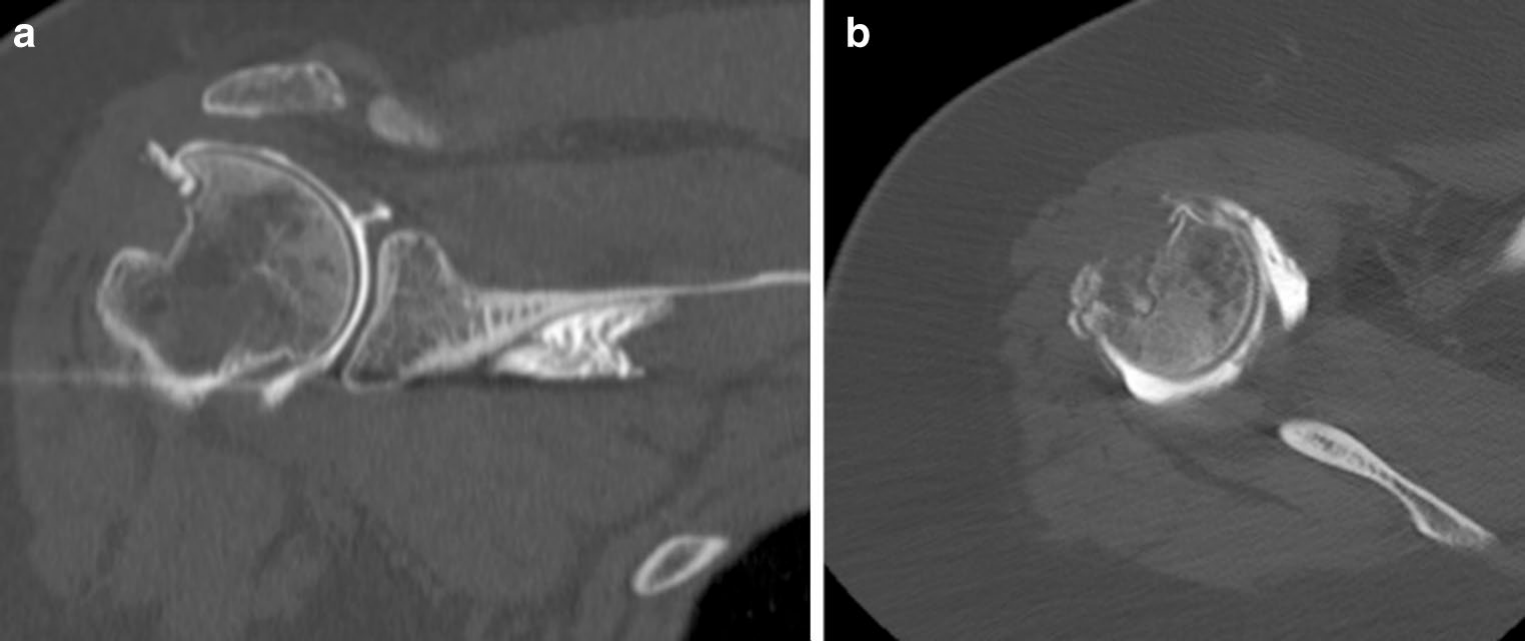
Preoperative coronal (**a**) and axial (**b**) CT scan demonstrating massive greater tuberosity bone loss

A 44-year-old female office worker sustained a right anterior glenohumeral dislocation with an associated avulsion of the greater tuberosity, as described by Mutch et al. [13]. Open reduction and internal fixation of the fracture were performed with a tension band technique, using an anchor screw and nonabsorbable sutures [9]. The functional outcome was poor. The patient was painful and had marked weakness in external rotation and abduction. Active forward flexion and abduction were 40° with full passive motion, and active external rotation was 30°. Her single assessment numeric evaluation score [15] was of 40 %. Past medical history was significant for noninsulin-dependent diabetes mellitus and obesity (BMI 42). Radiographic evaluation demonstrated a complete osteonecrosis and resorption of the greater tuberosity (Fig. 1) with a suspicion of massive rotator cuff tear involving both supraspinatus and infraspinatus tendons, without retraction. There was a state 1 Goutalier fatty infiltration of the supraspinatus and infraspinatus muscles. The electromyographic evaluation of the axillary nerve was normal. It was decided to reconstruct the defects with a fresh-frozen Achilles allograft tendon with attached calcaneal bone. Written informed consent was obtained from the patient for publication of this case report and accompanying images.

### Surgical technique

**Fig. 2:**
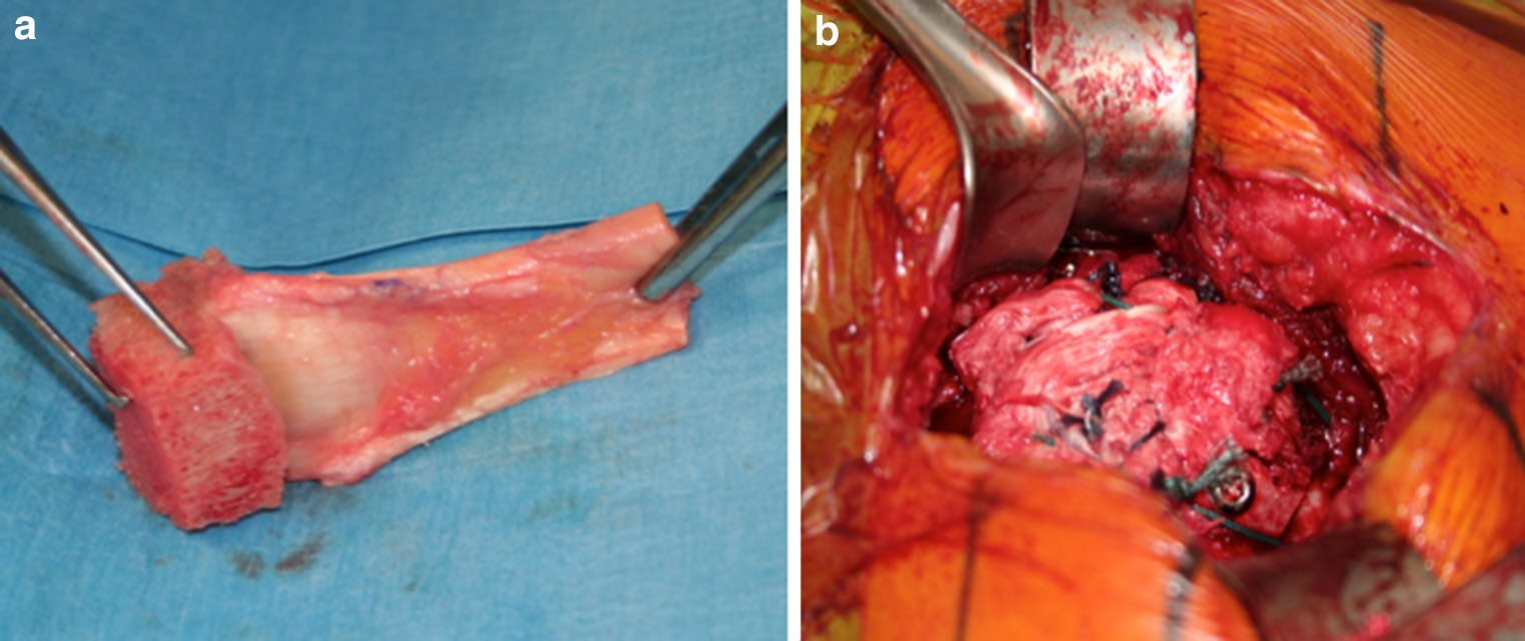
The allograft is prepared with an Achilles tendon split (**a**), which is inserted in order to reinforce the rotator cuff (**b**)

Under general anaesthesia and interscalene nerve block, the patient was placed in the beach chair position, with the operative arm draped free. An open anterosuperior incision with a deltoid split was performed in order to expose the greater tuberosity defect. The long head of the biceps was already resected. The remaining posterosuperior rotator cuff was carefully dissected, and the proximal humeral head was debrided. The quality of the cuff tissue was poor. The Achilles tendon allograft with attached calcaneus was then prepared (Fig. 2a). The calcaneus was shaped to fill proximal humeral head defect. The Achilles tendon was then split longitudinally, with the deep layer used to reinforce the rotator cuff repair and the superficial layer used to stabilise the allograft to proximal humeral diaphysis. Then, the bony portion of the allograft was secured to the humeral head with a 4-mm malleolar screw under fluoroscopic control. The native rotator cuff was then repaired onto the bony graft with a combination of an anchor (Haelix^®^, DePuy Mitek, Inc., Raynham, MA) and bone tunnels in the graft. The deep split of Achilles tendon was then sewn into the native posterosuperior rotator cuff to reinforce the repair (Fig. 2b). Finally, the superficial Achilles split was return on itself and attached laterally with another anchor (Haelix^®^, DePuy Mitek, Inc., Raynham, MA) in order to stabilise and cover the allograft.

**Fig. 3:**
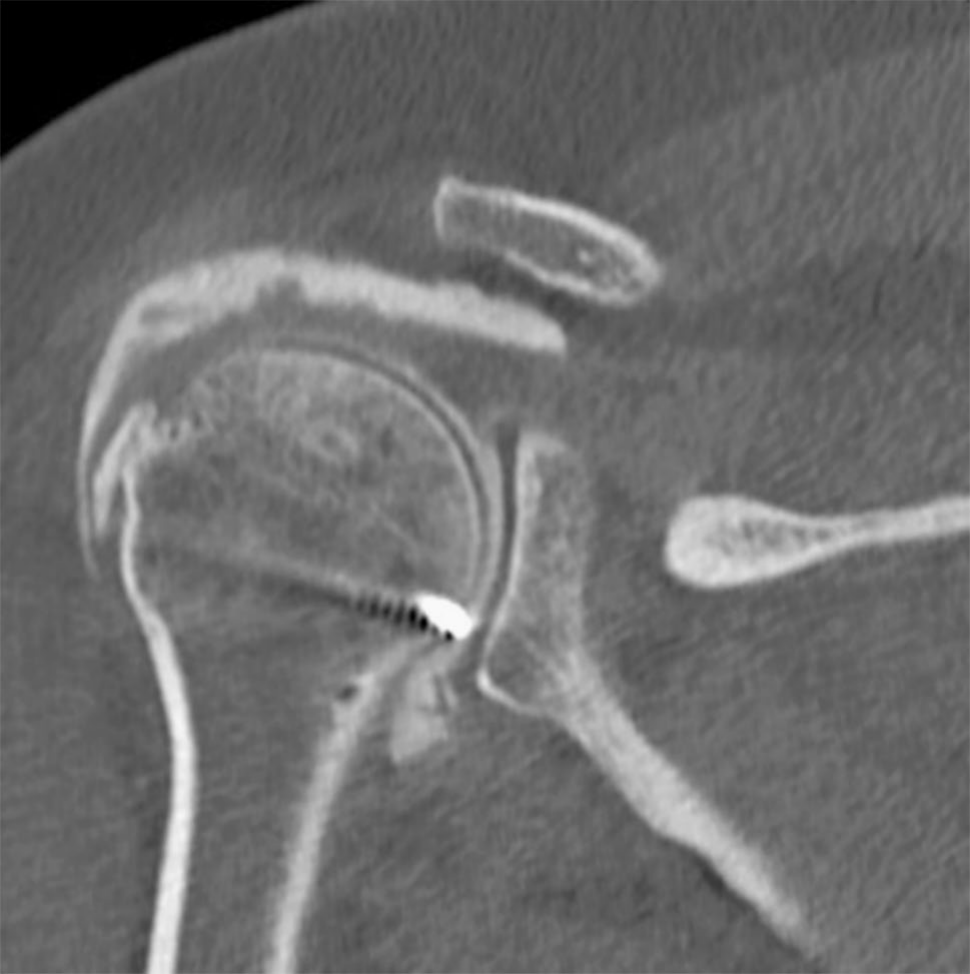
Postoperative arthroscanner shows the bony integration of the Achilles-calcaneal allograft, and a good integration of posterosuperior tendinous graft

Postoperatively, the patient wore an abduction sling for 6 weeks and was allowed to perform pendulum exercises only. Passive mobilisation was performed from weeks 6 to 12. Then, strengthening with progressive loads was initiated, and the patient returned to full activities level. At 6- to –12-month follow-up, she was pain free and recovered a full active range of motion compared to the opposite side, with an active antepulsion of 150° and an active external rotation of 50°. The SANE score was of 95 %. On plain radiographs, the bony portion of the allograft was integrated with the proximal humerus. The arthroscanner showed a good integrity of the tendinous graft (Fig. 3).

## Discussion

This case illustrates that an Achilles tendon allograft with attached calcaneus may be a surgical option in the surgical management of young patients with a combined posterosuperior rotator cuff tear and greater tuberosity deficiency. The clinical and radiographic outcome was excellent.

During repair of a massive rotator cuff tear, anatomical restoration of the cuff is always the goal as complete repair is associated with a better functional outcome [1, 7, 10]. However, this is not always possible with native tissue in the setting of poor tissue quality and bony defects. A large bony defect in particular significantly lowers the prognosis for primary repair [12]. One explanation might be that deltoid tension and therefore function are potentiated by the greater tuberosity, also called “deltoid wrapping” [14]. Therefore, reconstruction of the combined problem as in this case report may require both tendinous and bony reconstruction. Achilles tendon allografts have previously been reported in the reconstruction of combined tendon and bone deficiencies elsewhere in the body such as the elbow for olecranon and triceps deficiency [3]. Bony allograft has also demonstrated potential in the management of greater tuberosity defects [5] and humeral head osteonecrosis following rotator cuff repair [8]. Combined bony and tendinous shoulder allografts have also been proposed for reconstruction in the setting of malignancy or reverse shoulder arthroplasty using proximal humerus head allograft and attached tendons stumps [4]. In a series of 20 patients with proximal defects following resection of a malignant tumour, 16 recovered shoulder function, but only one with full range of motion. The current case report provides further evidence that fresh-frozen allograft is a viable option for combined bony and tendon deficiency. However, long-term follow-up is lacking, and the favourable outcome observed should be confirmed with future case series. Despite this, the technique presented here offers an opportunity to perform an effective conservative surgery for well-selected young patients with shoulder pseudoparalysis.

## Conclusion

Achilles tendon allograft might be a surgical option in management of a combined massive rotator cuff tear and greater tuberosity deficiency.
